# Selective Partitioned Regression for Accurate Kidney Health Monitoring

**DOI:** 10.1007/s10439-024-03470-8

**Published:** 2024-02-27

**Authors:** Alex Whelan, Ragwa Elsayed, Alessandro Bellofiore, David C. Anastasiu

**Affiliations:** 1 Computer Science and Engineering, Santa Clara University, 500 El Camino Real, Santa Clara, CA 95053 USA; 2 Biomedical Engineering, San José State University, 1 Washington Sq, San Jose, CA 95192 USA

**Keywords:** Point-of-care testing, Estimated glomerular filtration rate, Serum creatinine concentration, Color space, Histogram of colors

## Abstract

The number of people diagnosed with advanced stages of kidney disease have been rising every year. Early detection and constant monitoring are the only minimally invasive means to prevent severe kidney damage or kidney failure. We propose a cost-effective machine learning-based testing system that can facilitate inexpensive yet accurate kidney health checks. Our proposed framework, which was developed into an iPhone application, uses a camera-based bio-sensor and state-of-the-art classical machine learning and deep learning techniques for predicting the concentration of creatinine in the sample, based on colorimetric change in the test strip. The predicted creatinine concentration is then used to classify the severity of the kidney disease as healthy, intermediate, or critical. In this article, we focus on the effectiveness of machine learning models to translate the colorimetric reaction to kidney health prediction. In this setting, we thoroughly evaluated the effectiveness of our novel proposed models against state-of-the-art classical machine learning and deep learning approaches. Additionally, we executed a number of ablation studies to measure the performance of our model when trained using different meta-parameter choices. Our evaluation results indicate that our selective partitioned regression (SPR) model, using histogram of colors-based features and a histogram gradient boosted trees underlying estimator, exhibits much better overall prediction performance compared to state-of-the-art methods. Our initial study indicates that SPR can be an effective tool for detecting the severity of kidney disease using inexpensive lateral flow assay test strips and a smart phone-based application. Additional work is needed to verify the performance of the model in various settings.

**Supplementary Information:**

The online version contains supplementary material available at 10.1007/s10439-024-03470-8.

## Introduction

Chronic kidney disease (CKD) is a major cause of death globally [1]. In the US, it is estimated that 37 million people have CKD [2]. CKD progresses through several stages, each associated with a more severe loss of kidney function, causing the accumulation of toxic waste in the bloodstream. With early diagnosis and treatment, it is possible to slow or stop the progression of kidney disease [3]. However, because the early stages of CKD are generally asymptomatic, over 90% of CKD patients are not aware of their disease [4]. The lack of symptoms in the early stages of CKD leads to unacceptably high rates of late diagnosis, which is associated with worse prognosis [5]. Preventative strategies to improve CKD prognosis require early detection, especially screening for high-risk subjects, and frequent monitoring of patients with kidney function impairment [3, 6]. The current diagnostic guidelines for CKD require the persistence of abnormal markers of kidney dysfunction for at least 3 months [7]. These markers are low levels of the creatinine-based estimated glomerular filtration rate (eGFR, which should be lower than 60 mL/min/1.73 m^2^) and elevated albumin-to-creatinine ratio (ACR, which should be at least 30 mg/g). Recently, cystatin C has been proposed as an additional marker to calculate eGFR [8–10]. The high cost of clinical lab testing for those markers affects the frequency of testing among the lower income populations, making early detection in high-risk individuals less likely. In addition, the lack of rapid, inexpensive tests for CKD screening and monitoring hinders accurate assessment of CKD severity, progression and response to treatment, and for prompt adjustments of medication dose.

Creatinine is a waste product of muscle metabolism. Kidneys are tasked with filtering almost all the creatinine from the blood and releasing it into urine. When kidney function is impaired, creatinine levels in blood serum are abnormally high. Elevated creatinine levels in blood serum, or serum creatinine, is an established indicator of poor kidney performance [11]. While there is some disagreement about the most suitable physiological markers and most accurate analytical techniques [12–14], in routine clinical tests serum creatinine concentration is generally measured via colorimetric detection, and the result is plugged into an empirical equation to calculate the eGFR. The color change is produced by the chemical reaction of creatinine with either picric acid (Jaffe reaction) or an appropriate enzyme. The modification of diet in renal disease (MDRD) and chronic kidney disease epidemiology collaboration (CKD-EPI) are the two empirical equations commonly used clinically [15]. Reference ranges of eGFR values allow to diagnose CKD and classify disease severity.

Point-of-care testing (PoCT), usually performed by the patient at home, has been successful in the screening and management of acute and chronic conditions, such as COVID-19, hypertension and diabetes [16, 17]. The adoption of robust and cost-effective solutions for CKD monitoring, combining point-of-care testing devices and the recent advances in digital health, could dramatically improve clinical outcomes. However, the few options available are either too expensive (i-STAT handheld device by Abbott) or inaccurate (StatSensor Creatinine device by Nova Biomedical) [18, 19]. To date, no point-of-care testing device for CKD has been approved for home use [17].

**Fig. 1:**
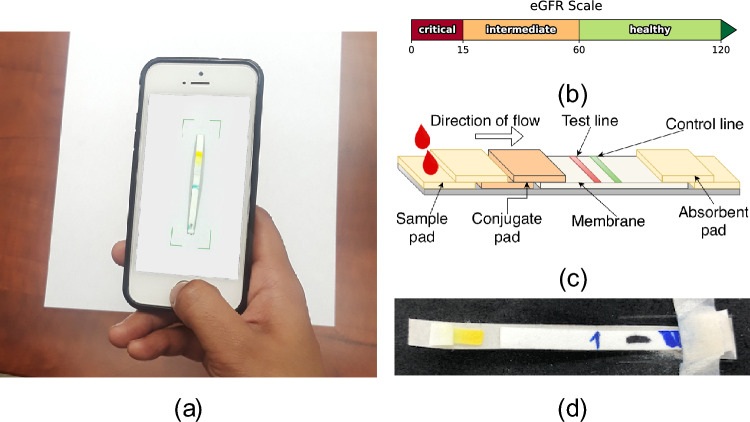
**a** An example kidney health monitor (KHM) application that can be used to predict the severity of kidney disease by analyzing the image of a test strip. **b** The estimated glomerular filtration rate (eGFR) range boundaries used in classifying the progression of CKD. **c** An example lateral flow assay test strip design. **d** An example test strip from our initial experiments. The detection zone changes color from an initial cream to yellow (as in the example) or reddish orange depending on the amount of creatinine in the sample

The overarching goal of our research is to develop a novel platform for accurate and affordable point-of-care testing of serum creatinine. We propose that such platform can be achieved by combining an inexpensive test strip (such as lateral flow assay, or LFA), a smartphone camera, and machine learning models. A single blood droplet, collected via finger-prick sampling, is exposed to an appropriate reagent on the LFA device to produce a colorimetric response. An image of the reaction pad on the test strip, captured by the patient’s smartphone, is analyzed by a machine learning model to estimate GFR based on the color change.

Several approaches have been proposed for low-cost point-of-care testing, including for CKD [20, 21]. The use of smartphone cameras for measuring colorimetric responses is also not new [22–26], and neither is the use of machine learning techniques for image processing, from simple tasks such as object detection [27] to advanced algorithms used for traffic analytics [28]. However, only a handful of studies have combined low-cost testing devices with AI to increase accuracy. Thakur et al. [29] designed a PoCT system for CKD screening by measuring urine albuminuria and performed classification on 10 discrete concentrations. They achieved an accuracy of 92% using the RF algorithm on samples taken in constant illumination. Solmaz et al. [30] developed a smartphone application called “ChemTrainer” that utilizes paper-based chemical assays for predicting hydrogen concentration using SVM and RF. “DeepLactate” [31] is yet another mobile application which measures lactate levels in sweat using a wearable bio-sensor. The app utilizes a vast array of embedded deep learning models for feature extraction and prediction including MobileNet [32], Xception [33], and VGG [34], among others. Bio-monitoring devices such as wearable lateral flow assays (LFAs) [35] and dermal tattooing biosensors [36] still require a mobile device to capture and analyze colorimetric change. Unlike our proposed approach, colormetric analysis systems designed by Roda et al. [37] and Zangheri et al. [38] involve the use of external devices or phone attachments.

In this paper, we focus our efforts on the ability of machine learning models to automatically predict the severity of kidney disease simply by analyzing the color of the detection zone of a test strip. Towards that end, we present a novel machine learning model, named selective partitioned regression (SPR), which is used to predict the severity of kidney disease as healthy, intermediate, or critical, using data from a smartphone camera. An initial classifier is used to do a course-grained prediction of the sample’s creatinine range, which is then followed by using a regressor trained on samples in that specific range to predict a more precise creatinine concentration. The final classification is obtained by eGFR thresholding, as illustrated in Fig. 1b.

## Materials and Methods

Our ultimate goal is creating a simple and inexpensive kidney function screening system that can be used at home as a preliminary indicator of kidney health. Towards that end, we have designed a cell-phone based kidney health monitoring application, which we describe in the "The Kidney Health Monitor Application" section, that automatically focuses on and scans a test strip we designed to react colorimetrically to the creatinine in the sample, and then uses machine learning models to predict the severity of kidney disease. In the "Test Strip Design" section through the "Test Conditions" section, we describe the design of the LFA test strip, the test fluid, image capture and test conditions. In the "Sample Augmentation" section, we describe the sample augmentation strategies we considered and our choice to increase the size of our data set. Finally, the "Selective Partitioned Regression" section describes the machine learning models we have designed to translate the color of the test strip detection zone to the kidney health status.

### The Kidney Health Monitor Application

**Fig. 2:**
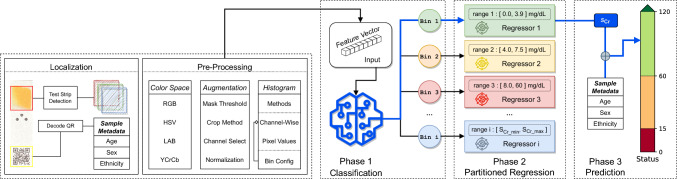
The full end-to-end pipeline used by our KHM application, including the architecture for our selective partitioned regression (SPR) model. In the *Localization* stage, the application first identifies the location of the detection zone of the test strip and extracts its pixels. Then, during *Pre-Processing*, the pixel values are transformed into a numeric *feature vector* given a chosen color space, augmentation, and feature type, based on SPR meta-parameter choices. The *feature vector* is first used to identify a bin, who’s associated trained regression model is then used to predict the amount of creatinine in the sample. Finally, taking in consideration the person’s age, sex, and ethnicity, the eGFR value is computed for the sample and the final kidney health prediction status is displayed.

To aid in the early detection of kidney health problems, we developed a kidney health monitor (KHM) application [39], which allows us to capture quality images of a sample test strip and immediately perform inference using a locally stored machine learning model to detect the severity of CKD. Our proposed solution aims at offering continuous monitoring as a PoCT service. Our goal is to be able to offer the KHM system to under-developed regions of the world that suffer from CKD but have no means of obtaining continuous testing.

Figure 2 showcases the full end-to-end pipeline used by our KHM application, including the architecture for our selective partitioned regression (SPR) model, which is detailed in the "Selective Partitioned Regression" section. In the *Localization* stage, the application first identifies the location of the test stip detection zone and extracts its pixels. During *Pre-Processing*, the pixel values are transformed into a numeric *feature vector* given a chosen color space, augmentation, and feature type, based on SPR meta-parameter choices. The *feature vector* is then used to predict the amount of creatinine in the sample via a pre-trained SPR model. Finally, the eGFR value is computed taking in consideration the person’s age, sex, and ethnicity. Additional details about the application can be found in Whelan et al. [39].

### Test Strip Design

LFA test strips were assembled using cellulose fiber sample pads and glass fiber diagnostic pads. A pressure-sensitive adhesive layer holds all paper pads together. An opaque plastic sheet covers part of the adhesive layer for user handling. A droplet of the test fluid is absorbed onto the sample pad and diffuses through the diagnostic pad. Preliminary tests focused on selecting appropriate dimensions for the sample pad (0.5 $\times$ 0.5 cm), diagnostic pad (0.3 $\times$ 1.0 cm) and pressure-sensitive adhesive layer (0.5 $\times$ 6.4 cm). A 0.25 cm overlapping of the sample pad over the diagnostic pad proved to be effective for diffusion to the reaction area. The diagnostic pad is pre-treated with 5 $\upmu$l an alkaline solution of picric acid (1 part of 0.04 M picric acid, two parts of 2.0 M NaOH solution), which reacts with the creatinine in the test fluid and produces a colorimetric response (Jaffe reaction).

### Test Fluid

Since the primary focus of this study is to evaluate the performance of our machine learning models, a simplified approach was chosen in this study to obtain a dataset of images with colorimetric responses at known creatinine concentrations $S_{Cr}$. Specifically, we used a synthetic creatinine solution to create our dataset, rather than human or human-analog blood. Samples of test fluid at 65 different creatinine concentrations were prepared starting from 9 mg of creatinine powder dissolved in 15 ml of HCl solution (0.1 M), which yielded the highest creatinine concentration of 60 mg/dl, and then proceeding with serial dilutions all the way down to 0.1 mg/dl.

### Image Capture

In this study, images were collected under controlled lighting conditions. The test strips were placed inside a 40 $\times$ 40 $\times$ 40 cm lightbox equipped with LED light sources and lined with reflective materials to eliminate the environmental light variation and ensure uniform illumination. Images of the test strip against a black background were collected using a 12 MP smartphone camera (Apple iPhone 8, Cupertino, CA) placed at a fixed distance of 8 cm from the strip. That focal distance was selected as the best tradeoff between autofocus accuracy and image magnification. The smartphone was set on auto-brightness (with 0-ev exposure compensation) and output uncompressed images in PNG format, which preserved more color information than the more common compressed JPEG format. The resulting images are 3024 $\times$ 4032 pixels and take up approximately 15 MB of storage. The samples were manually cropped and annotated using BBox-Label-Tool [40] and OpenCV library functions [41]. The final annotated detection zone samples have a 2:1 height-to-width aspect ratio and are each approximately 256 $\times$ 128 pixels.

### Test Conditions

Based on the MDRD equation, eGFR appears to be most sensitive to creatinine concentration in the range 0–4 mg/dL. Thus, we designed our experiments to collect data in the ranges and steps detailed in Table 1, prioritizing sample collection on the low end of the distribution. We captured 3 test strip image samples per concentration step, before adding the creatinine solution to the the detection zone, and then at intervals of 2, 12, and 22 min after adding the creatinine solution. That resulted in a total of 780 samples. Fig. 1 and 2 in Section 1 of our Technical Appendix illustrate the change in color saturation and brightness of the samples as time passes.

**Table 1:** Distribution of samples according to bin ranges

Range (mg/dL)	Steps (mg/dL)	Concentrations	# Samples
$0.0-4.0$	0.1	41	492
$4.5-7.5$	0.5	7	144
$8.0-19.0$	1.0	12	84
$20.0-60.0$	10.0	5	60

### Sample Augmentation

**Fig. 3:**
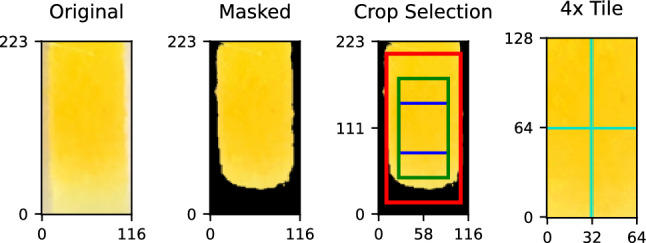
Pre-processing and sample augmentation pipeline. The *Original* image contains all pixels of the identified detection zone of the test strip. The *Masked* image shows the image after removing pixels whose color is outside our expected ranges. The *Crop Selection* image shows a variety of cropping strategies we have used to ensure quality inputs for our machine learning pipeline. Ultimately, we chose to use a 128 $\times$ 64 center-crop strategy, exemplified by the green rectangle. The final image, labeled *4x Tile*, shows how the chosen 128 $\times$ 64 center crop image is further sub-divided into 4 equal size tiles, which allows us to create 4 samples from each executed chemical experiment.

Due to the time and financial costs of chemical experiments, our initial dataset is limited in size. Therefore, we performed data augmentation, which allowed us to increase the number of samples we could use to train our machine learning models. Figure 3 illustrates our pre-processing pipeline utilized in obtaining quality samples. The first image shows the detection zone from a random sample. We begin by threshold masking the image using the HSV color space in order to remove the background and any abnormal pixel values that may have been introduced during the data collection or chemical reaction phase, as these might be detrimental to our model’s predictive performance. The second image in Fig. 3 shows the detection zone after masking. Based on our earlier color change analysis depicted in Fig. 1 and 2 in the Technical Appendix, we noticed that the change observed in the Jaffe reaction belongs to a particular range of colors, namely yellow to orange, which we used to obtain our masking thresholds. Our chosen ranges were $\left[ 20, 60\right]$, $\left[ 150, 255\right]$, and $\left[ 230, 255\right]$ for the hue, saturation, and value channel pixel values, respectively, in the HSV color space. Next, in order to avoid background pixels, we center-cropped the detection zone to a size of 128 $\times$ 64, as depicted in the third image in the figure. Finally, we partitioned the 128 $\times$ 64 image into four equal-sized 64 $\times$ 32 tiles, which form the new samples we use in our experiments. After augmentation, our dataset size increased from 780 to 3120 samples.

The reason we chose to extract a 128 $\times$ 64 center crop of the detection zone as part of our processing pipeline is that some of our baseline models require input images to have a constant size and we wanted to ensure all models were trained and evaluated based on the same inputs, not only based on the same image samples. We also considered random sampling for the third step in our pipeline, which would potentially allow us to create more than just 4 samples for each detection zone capture, but that technique would provide an advantage to some methods due to the pixel overlap in the samples that would undoubtedly occur. Another common augmentation technique from digital image processing is the application of smoothing filters, which alter pixels based on a non-linear function of their neighborhood pixel values. This technique is often used to smooth abnormal or extreme values that may be introduced due to artifacts or noise in the image. However, because we are dealing with interpreting very slight colorimetric changes, any form of color interpolation that affects the ground truth of the sample should be avoided.

### Selective Partitioned Regression

We propose a novel machine learning model that we call the Selective Partitioned Regression (SPR) model. Our model architecture, which is illustrated in the right section of Fig. 2, is segmented into two phases and uses a composition of specialized state-of-the-art algorithms as sub-estimators that perform classification and regression. The first phase of our model is a classification task that involves selecting a particular localized regressor that is trained on a subset of partitioned samples. The second phase is a regression task where we use the predicted regressor to estimate the concentration of creatinine present in the sample. The final classification decision is made based on the estimated glomerular filtration rate (eGFR), which is computed using the MDRD equation [42] and defined as

1 $$\begin{aligned} eGFR&= 175 \times S_{Cr}^{-1.154}\ \times \text {~age}^{-0.203}\ \times 0.742~\text {~if~female}\nonumber \\&\quad \times 1.212~\text {~if~African~born}, \end{aligned}$$

where $S_{Cr}$ is the creatinine concentration in the sample, measured in mg/dL. For a given patient, all metadata such as age, sex, and race in the formula are static, while the creatinine concentration, which is the value we are predicting, may change over time. Using the boundary threshold values from Fig. 1b, we can classify an individual’s kidney disease severity as healthy, intermediate, or critical. This is consistent to works done by Wei et al. [43], Ronco et al. [44], and Chawla et al. [45], among others.

The performance of our model is affected by several choices, including the color space that we represent our input images in, the type of features we extract from the images, the boundaries of the local regressor bins, and the sub-estimators we choose for the classification and regression tasks. In the remainder of this section, we describe each of these choices in detail.

#### Color Spaces

Images are traditionally represented in the red-green-blue (RGB) color space, which represents the amount of red, green, and blue that should be mixed to create the color of a pixel, each in the range from 0 to 255. A 64 $\times$ 32 pixel image is thus represented as a 64 $\times$ 32 $\times$ 3 tensor containing the red, green, and blue values for each of the image pixels, which is often reshaped into a vector by concatenating each channel of each row of pixels in the image. In the RGB representation, the channels hold both the chrominance and luminescence information, making the extracted features susceptible to change given even small brightness variances. Several color spaces, including LAB, HSV, and $\text {Y}\text {C}_r\text {C}_b$, treat luminescence as a separate channel, which we hypothesize can improve our model’s predictive ability when representing pixels in these color spaces vs. RGB.

The LAB color space was devised by Richard S. Hunter and formalized in the Photoelectric Color Difference Meter [46] article in the Journal of Optical Society of America in 1958. It is an almost perceptually uniform color space aimed at mimicking the human vision system. The L component stands for lightness and ranges from [0, 1], or from the absence of light (black) to complete illumination (white). The *a* and *b* components, often abbreviated as $a^*$ and $b^*$, range from $[-127,127]$ and represent opposing colors, e.g., $\left( \text {green} \rightarrow \text {red}\right)$ and $\left( \text {blue} \rightarrow \text {yellow}\right)$, respectively. The $a^*$ and $b^*$ axes are perpendicular to each other on the color wheel and there is a $180^{\circ }$ difference between their respective positive and negative ends. Sometimes the $a^*$ and $b^*$ opposing colors are represented as $\left( \text {green} \rightarrow \text {magenta}\right)$ or $\left( \text {cyan} \rightarrow \text {yellow}\right)$, depending on how the axes are aligned with regards to their initial position.

The Hue-Saturation-Value (HSV) color space, also known as the HSB (hue, saturation, brightness), models how colors appear under light. The hue channel ranges from [0, 360] representing the angle from which the chroma is selected. Saturation and value both range from [0, 1], where 0 denotes complete saturation and the absence of illumination (black) for the value channel. The value 1 denotes maximum chroma, which maximizes the perception of the pixel hue.

Lastly, the Y in Y$\text {C}_r\text {C}_b$ stands for luma or luminance/illumination, and $\text {C}_r$ and $\text {C}_b$ are complements of Y with red and blue, i.e., (red - Y) and (blue - Y). Y$\text {C}_r\text {C}_b$ is derived from RGB but was devised to be an approximation of a perceptually uniform color space. The advantage of using Y$\text {C}_r\text {C}_b$ over the other color spaces is that it requires far less storage to encode the same information as RGB, which makes it a perfect candidate for use in color processing pipelines used by digital systems that require high bandwidth and throughput.

#### Feature Extraction

We focused primarily on two methods for feature extraction, channel-wise histograms and raw pixel values. First, we can construct a histogram representation of an image by binning the pixel values from a selected range across the three possible channels and concatenating them together. We refer to this feature extraction method as *channel-wise histogram of colors* (HOC). The other method of extracting features is simply using the normalized channel-wise pixel values of the image remapped to be in the same range $\left[ 0,255\right]$ across all color spaces, which we refer to as the *pixel values* feature extraction method.

Most image processing libraries perform pixel normalization and remapping implicitly but they are not always consistent. For example, in the case of the LAB color space, some libraries will express the $a^*$ and $b^*$ in the range of $[-127,127]$, while others remap the values to be in the range [0, 255] or $[-1,1]$. The general formula used for remapping pixel values from $S \mapsto T$ is given by

2 $$\begin{aligned} T_k = \frac{\left( S_i - S_{min}\right) ~\cdot ~\left( T_{max} - T_{min}\right) }{\left( S_{max} - S_{min}\right) } + T_{min} \end{aligned},$$

where *S* is the input pixel range, *T* is the output pixel range, $T_k$ is the transformed pixel value in the output range, and $S_i$ is the pixel value in the input range.

**Fig. 4:**
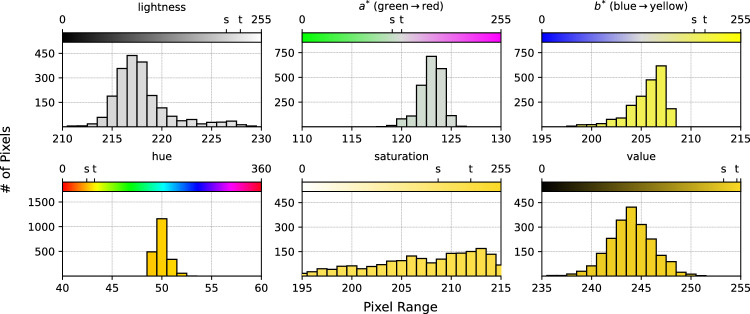
Comparison of the pixel distribution from the LAB (top) and HSV (bottom) color spaces represented as binned channel-wise histogram of colors (HOC). The components of each color space are shown in each figure along with the distribution of values in our collected samples for each component. While the top band in each figure shows the full range of values across the entire color spectrum, our test strips contain a limited range of colors and the distributions are focused on the subset of values between the *s* and *t* marks on each band.

**Histograms** A histogram of colors is the frequency of occurrence of pixel values in a given dataset or image. It may also be interpreted as an approximation to a probability distribution function if we normalize the histogram by dividing by the total number of non-masked pixels in an image. To demonstrate the computation for the histogram of colors, we use the masking thresholds from the HSV color space we noted in the "Sample Augmentation" section to represent the hue ($\alpha$), saturation ($\beta$), and value ($\gamma$) channels, for the sampling ranges (R) are defined as $R_\alpha = [20,60]$, $R_\beta =[150,255]$, and $R_\gamma =[230,255]$. A channel-wise histogram with *K* bins for each channel will record the number of pixels (excluding the masked pixels) whose $\alpha$, $\beta$, and $\gamma$ values fall within that range giving us a total of $K_{\alpha ,\beta ,\gamma } = [40,105,25]$ maximum number of bins per channel. Figure 4 shows a comparison of the pixel distribution from the LAB (top) and HSV (bottom) color spaces represented as binned channel-wise histogram of colors (HOC) in our method, based on our experiment data.

**Pixel Values and Convolutions** The pixel values of an image can be considered features on their own. However, using the normalized channel-wise pixel values we can also perform convolutions on the image to extract rich feature maps in our deep learning models. Channel-wise convolution operations are generically defined as

3 $$\begin{aligned} I(x,y) = \sum _{s=s_1}^{s_2} \left( \sum _{t=t_1}^{t_2} h(s,t) \times I(x-s, y-t)\right) , \end{aligned}$$

where *h*(*s*, *t*) is the kernel for the convolution filter and *I*(*x*, *y*) is a single channel image. Each output pixel is the weighted sum of the input pixels with the kernel function.

A benefit of using histograms over pixel values is that the feature vector length is significantly smaller, which can reduce processing time and increase throughput, thus making our system more scalable. For example, a single tiled $64 \times 32$ sample in our data results in a 765-value feature vector using the HOC representation, but requires 6, 144 features for its pixel-based representation.

#### Partitioning

**Fig. 5:**
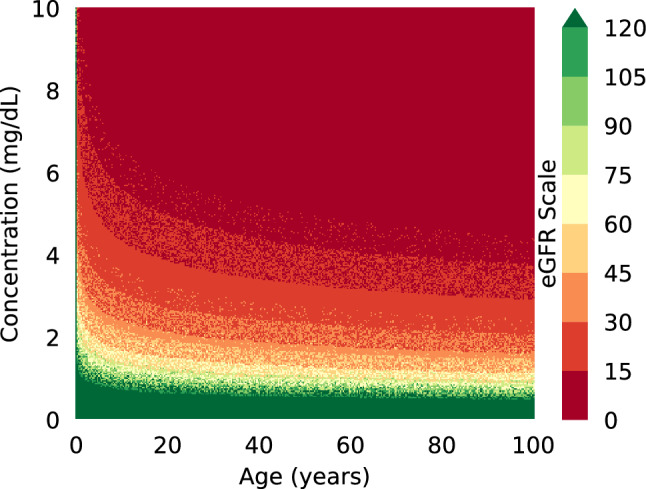
Predicted eGFR values distribution for random samples in which the creatinine concentration was uniformly randomly sampled and “people” were randomly selected according to the 2019 US census population statistics. The eGFR value is denoted by the color of the dot. The snow-like effect of mixed dots of different nearby colors shows that the sample contains different people with very similar ages for which the kidney disease severity outcome is different.

The range of creatinine values that our regression model must predict is quite large and the values are not linearly spread out across the range. Moreover, since the regression predictions are used to classify the severity of kidney disease using Eq. 1, it is important that predictions around the decision boundaries between *critical* and *intermediate* and *intermediate* and *healthy* be as accurate as possible. However, the eGRF formula is also influenced by other factors, such as sex, age, and race. To showcase this, Fig. 5 shows eGFR values for a large number of tests in which the creatinine concentration was uniformly randomly sampled and the “people” the concentration belonged to were randomly selected according to the 2019 US census population statistics [47]. To simplify viewing, we only show the concentration and age values on the heatmap graph, and the eGFR value is denoted by the color of the dot. The sex and race of the person are however accounted for in the eGRF calculation. Note that a creatinine concentration of 5 mg/dL leads to a *critical* diagnosis for most people above 50 but only *intermediate* for younger people. Also note the snow-like effect in the image denoted by the difference in outcome that age, sex, and race play in the eGRF computation. Several bands in the graph have mixed red and orange dots or green and yellow dots, denoting different people with very similar ages for which the kidney disease severity outcome is different.

Based on these analyses, we hypothesize that partitioning the samples into multiple bins and training separate regression models for each bin can lead to improved eGFR classification effectiveness. However, we do not know which bin a new sample belongs to. Therefore, as shown in the architecture design illustrated in Fig. 2, our SPR model consists of two stages, first a classification phase for predicting the bin the sample belongs to, followed by the regression stage where we use the selective regressor trained for that bin to predict the creatinine concentration $S_{Cr}$. The predicted $S_{Cr}$ is then combined with the sample’s metadata to compute the eGFR value according to Eq. 1 and the severity of the kidney disease is then predicted based on the pre-defined boundary thresholds as *healthy*, *intermediate*, or *critical*.

The questions that remain to be answered with respect to partitioning are how many partitions we should have and what should be the ranges of those partitions. Our model is parametric with respect to the number of partitions/regressors $\chi$ and the granularity of the concentration ranges $\delta$ and we discuss several choices for these parameters in the "Results" section.

#### Sub-Estimators

Our SPR model uses existing state-of-the-art predictors that were also found to be effective in similar colorimetric problems described in the "Discussion" section, namely Histogram Gradient Boosting Decision Tree (HBT) [48], Extreme Gradient Boosting Tree (XGB) [49], Random forest (RF) [50], K-Nearest Neighbors (KNN) [51], Decision Tree (DT) [52] and support vector machines (SVM) [53]. We tested the classification and regression versions of each of these models for the two phases of our algorithm and report results in the "Results" section.

#### The Deep Neural Network Model

In addition to our SPR model, we also designed a deep neural network (DNN) model that is based on the VGG model [34] as the reference architecture. In order to reduce over-fitting, we updated the model with regularization techniques absent in the original VGG architecture, such as learning rate decay [54], early stopping [55], batch normalization [56], and drop-out [57]. The model uses a 5-layer convolutional neural network (CNN) architecture with incrementally increasing filter sizes, reduced by a factor of 2 compared with VGG, i.e., 32, 64, 128, 256, and 256, using LeakyReLU activations with $\alpha ={1e-3}$ instead of ReLU. Additionally, our model performs batch normalization before max pooling. The convolution network is then joined with two fully connected layers of size 4096 where drop-out regularization is applied with a $25\%$ probability before finally reaching the linear activation function that returns the regression result predicting the creatinine concentration $S_{Cr}$. We used the Adamax [58] learning rate optimizer to train this model with an initial learning rate of $\lambda ={1e-4}$, a variation of the Adam algorithm that is more suitable for time-dependent processes such as ours. The loss function used to calculate parameter updates was mean squared logarithmic error and we trained the network for 500 epochs in batches of 128 with a learning rate decay equivalent to $\lambda \cdot \text {e}^{{-5e-3}}$ and early stopping with a patience of 128 epochs.

## Results

In this section, we first describe the design of our experiments, including baseline algorithms, performance measures, validation methodology, and the execution environment we used for executing our experiments. Then, we detail the results of our experiments, along several directions. First, in the "Optimal Time Sampling" section, we identify the optimal time point for modeling kidney disease severity prediction and identify the best parameters for our novel 2-phase Selective Partitioned Regression model. Then, in the "SPR Parameter Choices" section, we analyze the effect that different parameter choices in our processing pipeline have on the performance of SPR and the top-performing baselines, including the choice of color space and the type of features being extracted. We then provide evidence in the "Baseline Comparison" section that our SPR model using multiple regressors outperforms state-of-the-art baselines, providing both high classification accuracy and low regression error. Finally, in the "Ablation Studies" section, we analyze the robustness of SPR with regards to several of its parameters, including the number of regressors $\chi$ and the granularity of the concentration ranges $\delta$.

### Experimental Design

#### Baseline Methods

Our state-of-the-art baselines were designed based on the methods proposed by Wei et al. [43], Ogunleye and Wang [59] and Thakur et al. [29]. They use pixel-wise feature extraction combined with the same regression algorithms we also use for our SPR sub-estimators, namely Histogram Gradient Boosting Decision Tree (HBT), Extreme Gradient Boosting Tree (XGB), Random Forest (RF), K-Nearest Neighbors (KNN), Decision Tree (DT) and Support Vector Machines (SVM). Figures and tables will refer to these algorithms by their abbreviated notation. The final classification decision is made in the same way as our method, by applying Eq. 1 and identifying the class the eGFR value belongs to according to the boundary thresholds. Unlike their original papers that used a given color space (such as LAB) during feature extraction, we tested each method with all the color spaces we described in the "Color Spaces" section and report results for each color space and compare our method against the best performing baselines across all color spaces.

#### Sample Assignments

In order to evaluate our model effectiveness, we assign each test strip sample an age, sex, and race consistent with Eq. (1). In this paper, we use the term *sample* referring to an image with a unique concentration, time point after applying the creatinine solution, and the accompanying metadata to be representative of a person. We used data from the 2019 United States Census Bureau [47] to define the population distribution that we sampled from. Table 2 summarizes the population percentages segmented by age, sex, and race that we used in our experiments.

**Table 2:** Age, sex, race sample distribution

	Percent (%)
Age (years)	
Under 5	6.1
5 to 24	25.6
25 to 44	26.5
45 to 64	25.4
65 to 84	14.6
Over 85	1.8
Sex	
Male	49.0
Female	51.0
Race	
African	13.4
Other	86.6

#### Performance Metrics

Due to the time and monetary costs of chemical experiments, our dataset is limited in size, even after applying our tiling procedure. Cross-validation of our model performance therefore becomes vastly more significant in determining how well our models generalize to unseen data. We performed 5-fold cross validation on all models using stratified sampling based on the patient’s CKD status. Stratified sampling ensures that the predicted classes, healthy, intermediate, and critical, have a proportional distribution in the training, validation, and test sets as observed across the entire dataset. In addition, since each sample is associated with a randomly selected “person”, we were able to repeat the experiment multiple times by re-sampling the population distribution and performing 5-fold cross-validation and test set inference during each experiment. We therefore report the mean performance across $N=10$ experiments with different populations drawn from the same distribution.

Model performance is based on *F1*-score for classification results and *RMSE* for regression results as the evaluation criteria. *RMSE* is computed as the square root of the sum of square deviations of the predicted values from the true values being predicted, i.e.,

$$\begin{aligned} RMSE = \sqrt{\frac{\sum _{i=1}^{n} \left( S_{{Cr}_{i}} - \hat{S}_{{Cr}_{i}}\right) ^2}{n}}. \end{aligned}$$

In our case, RMSE captures the deviation of the predicted $\hat{S}_{Cr}$ creatinine concentration from the ground truth creatinine $S_{Cr}$ concentration over the *n* test samples. *F1*-score represents the harmonic mean between precision and recall, i.e.,

$$\begin{aligned} F1 = 2\times \frac{\text {precision}\times \text {recall}}{\text {precision}+\text {recall}} = \frac{TP}{TP + \frac{1}{2}(FP + FN)} \end{aligned},$$

where *TP*, *FP*, and *FN* are the number of true-positive (correctly identified, i.e., predicted class is the same as the ground truth class), false-positive (identified incorrectly), and false-negative (not identified) samples, respectively.

While classification accuracy is the ultimate goal in this work, regression performance is also an important indicator that can tell how often a model may deviate from the correct class, especially for $S_{Cr}$ values that are close to the class boundaries for the given person. Therefore, as a way to evaluate methods based on both their classification and regression performance, we combined these metrics using a decision function that is a parametric weighted sum of the *F1*-score and *RMSE* values, computed as

4 $$\begin{aligned} f_\text {decision}(\alpha , \textit{F1}, \textit{RMSE}) = \alpha \cdot \textit{F1} + \frac{1-\alpha }{\textit{RMSE}} \end{aligned},$$

where $\alpha \in [0,1]$. While we separately analyze both regression and classification performance, we use our decision function with $\alpha =0.75$ as the main effectiveness measure when ranking all competing methods.

#### Execution Environment

All experiments were executed on a Linux workstation machine (Ubuntu 20.04 LST) with an an Intel^®^ Core^™^ i9-10900X 10 core CPU running at 3.70 Ghz, two Nvidia RTX 3090 GPUs (only one used for experiments) and 128 GB of memory. We designed our SPR model using the scikit-learn (ver. 1.2) custom estimator interface and our DNN model using the Keras (ver. 2.9) functional model interface.

### Optimal Time Sampling

The reaction of the creatinine in each sample with the picric acid solution in the test strip takes some unknown amount of time. As a result, when we executed the chemical experiments, we took pictures of each test strip at 2, 12, and 22 min after applying the creatinine solution. In a set of experiments we detail in Section 2 of our Technical Appendix, we compared the performance of all baseline models when trained on data from each time point and found that the 22 min time point provided the best classification and regression performance. In general, the worst results were achieved at 2 min, implying that 2 min is not enough time for the chemical reaction to give accurate results. Further experiments were then executed using only data from this time point. Moreover, we chose to eliminate the Support Vector Machines and Decision Tree algorithms from contention in further experiments as their performance was inferior compared to the rest of the available methods.

### SPR Parameter Choices

**Fig. 6:**
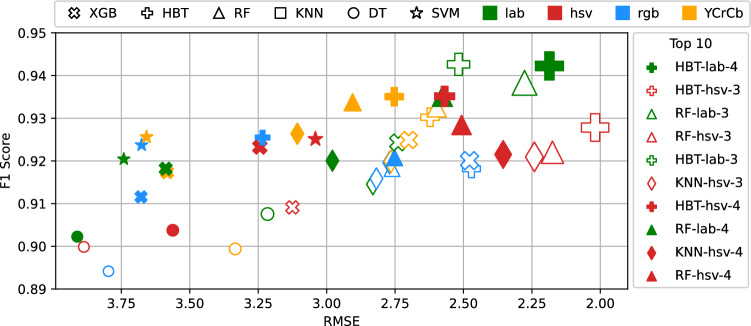
Evaluation of SPR model parameters using various sub-estimators (denoted by the bullet point shape), color spaces (denoted by color), and number of regressors $\chi$ (denoted by the number at the end of the label). Each model had its respective bin ranges $\delta$ tuned via cross-validation. The size of the bullet point is relative to the value of our decision function in Eq. 4. Values in the upper-right corner are better.

Our method is parametric with regards to the choice of color space, feature type, sub-estimators for regression and classification, the number of regressors, and the bin ranges for each local regressor. We tuned each of these parameters via cross-validation and chose the best performing combination based on our decision function denoted in Eq. 4. Figure 6 shows a subset of our experiment results while searching for the best parameters. In the figure, sub-estimator and color space choices are listed in the top key and denoted by the bullet point shape and color, respectively, and the number of regressors $\chi$ is denoted by the number at the end of the label in the Top 10 legend. Models had their bin ranges $\delta$ tuned via cross-validation. Bullet point size is relative to the value of our decision function. Values in the upper-right corner are better.

Overall, the model using 4 regressors, histogram of colors-based features extracted from the LAB color space, and the Histogram Gradient Boosting Decision Tree (HBT) sub-estimator achieved the highest decision function score. The model’s CKD classification *F1*-score was **0**.**9422** and the $S_{Cr}$ regression *RMSE* was **2**.**19**. Going forward, we use this version of our model in future comparisons with baselines.

Figure 6 shows results for matched sub-estimators, where the same sub-estimator algorithm was used for both phase-1 and phase-2 in our method. We also performed experiments with unmatched sub-estimators, i.e., using a different algorithm for the bin choice classification than the one used for the $S_{Cr}$ value regression, but did not find any significant improvement in the results.

The quality of our model is highly dependent on the effectiveness of the phase-1 classification model that chooses which local regressor should be used for the test sample during inference. We measured the performance of this classifier and report *F1*-score values in Table 3 for different sub-estimators and several different combinations of number of regressors and color spaces. Results show that our chosen best performing model also has the top bin classification performance across all tests.

**Table 3:** Bin classification performance

	XGB	HBT	RF	DT	KNN	SVM
Default (RGB)	0.9135	0.9240	0.9255	0.8755	0.8923	0.9062
3 Bin (HSV)	0.9327	0.9375	0.9303	0.9130	0.9193	0.9317
4 Bin (LAB)	0.9457	**0**.**9505**	0.9466	0.9034	0.9346	0.9375

Bold value represents the highest performing subestimator, i.e., the model using 4 regression bins, LAB color space features, and the Histogram of Boosted Trees regressor

### Baseline Comparison

**Fig. 7:**
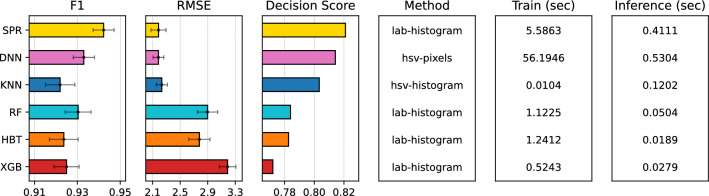
Comparison of the SPR model, using our decision function in Eq. 4, against the best performing baselines across all color spaces and feature types. Higher decision and *F1* scores and lower *RMSE* scores are better. Lower training and inference times are better.

Finally, we compared our chosen SPR configuration against state-of-the-art baselines noted in the "Baseline Methods" section. Each model had its respective meta-parameters tuned via cross-validation, e.g., the number of neighbors for *k*-nearest neighbors or the number of trees and maximum depth of each tree in the RF algorithms. For each baseline, we chose the best performing color space and feature type and we measured both model performance (via our decision function) and efficiency (via training and inference run-times). Figure 7 shows the comparison results, including the *F1*-score, *RMSE*, their respective errors across the 10 separate experiments that were executed (as error bars), and decision scores for each method. Note that, to increase visibility of the method comparison, only the top portion of the bars are shown and errors for most methods are actually quite small. The “Method” panel shows the best choice of color space and feature type for each method, while the right two panels report the training and inference times in seconds.

The results show a clear advantage of our SPR model. While achieving very similar *RMSE* (**2**.**19** vs. 2.18 and 2.23) as the next two alternatives, our SPR model is able to provide significantly better overall CKD classification performance, achieving an *F1*-score of **0**.**9422**. The optimal performance does come at the cost of inference efficiency as SPR incurs some overhead when using multiple regressors, however it is still able to significantly outperform the second most effective model, DNN, in terms of efficiency.

### Ablation Studies

In this section, we report the results of several ablation studies investigating the robustness of our method given different input meta-parameters. Due to lack of space, we summarize these results here and include an extended explanation and figures in our Technical Appendix.

#### Color Space Comparisons

As noted in the "Color Spaces" section, the chosen color space may play a big role in the performance of our model, as some color spaces separate luminescence from color representation while others do not. To see how the choice of color space in feature extraction affects model performance, we tested our model and all baselines with HOC-based features from each of the four color spaces described in the "Color Spaces" section, i.e, RGB, $\text {Y}\text {C}_r\text {C}_b$, HSV, and LAB. Overall results indicate that the LAB color space is beneficial for the classification task but the HSV color space is best for the regression task. It is interesting to note, however, that the color space performance of our SPR model was consistent between the classification and regression tasks (RGB is worst and LAB is best), while other methods saw almost complete inversions between the two tasks (e.g., RGB achieves the highest/best *F1*-score for DNN and also the highest/worst *RMSE* score).

#### Feature Type Comparison

Another choice in our method is pixel- vs. HOC-based feature extraction. We analyzed the performance of the best performing baselines and our SPR model using both types of features and results clearly indicate that HOC-based features are superior for the majority of the models in both the classification and the regression tasks.

#### Partition Parameterization

Our model performance also depends on the number of local regressors $\chi$ and the bin ranges $\delta$ selected for each local regressor. We tested both 3-bin and 4-bin configurations of our model with all color spaces and HOC-based features under a wide range of $\delta$ parameter choices. Overall, we trained 1, 250 models each for the 3-bin and 4-bin evaluations. Results show that, while the 4-bin LAB-based configuration achieved the best CKD classification performance, the 3-bin HSV-based configuration achieved the best regression performance, leading to better overall performance according to our decision function. Overall, however, the differences between the 3-bin and 4-bin configurations were small (0.9316 vs. 0.9422 F1-score and 1.93 vs. 2.19 RMSE), indicating that our model is resilient to the number of local regressors, as long as enough data exists to find appropriate bin boundaries and accurately train local regressors for each bin.

## Discussion

In this study, we introduced and evaluated a Selective Partitioned Regression model for kidney health monitoring, which uses images that a patient could capture at home using an inexpensive test strip, a single-droplet blood sample, and a smartphone. Our results showed that our SPR model is both effective and efficient in predicting the severity of kidney disease.

A crucial component of our testing platform is how the color map obtained from the reaction pad on the test strip is used to predict eGFR. To our knowledge, only few studies have attempted to associate color variations with a prediction outcome. One closely related system that predicts continuous values for color change observed in reaction between urine and picric acid solution is the homemade-spectrometer by Debus et al. [60]. Despite being a low-cost system, that setup requires technical expertise and is time consuming. In another study, Paulraj et al. showed that a 5 hidden layer artificial neural network could predict the ripeness of bananas with an accuracy of 96% [61]. Similarly, Loti et al. [62] used a vast array of deep learning models, such as VGG [34], ResNet [63], InceptionNet [64], and DenseNet [65], for feature extraction and classical machine learning algorithms, such as Support Vector Machines [53] (SVM) and Random Forest [50] (RF), for classifying diseases and pests in chili leaves. Their composite modeling structure is able to achieve a 92% accuracy using InceptionNet for feature extraction and SVM for classification.

Some researchers found that classical machine learning models sometimes outperform deep learning ones in vision-based biomedical problems, while others found the opposite. Daghrir et al. [66] compared K-Nearest Neighbors (KNN), SVM, and Convolution Neural Networks (CNN) when classifying whether detected melanoma skin cancer was malignant or benign and obtained accuracy values of 57.3%, 71.8%, and 85.5%, respectively. On the other hand, both Wei et al. [43] and Ogunleye and Wang [59] found XGBoost [49] to have superior predictive performance in CKD severity classification over other algorithms such as Logistic Regression (LR), SVM, and even Artificial Neural Networks (ANN). While these works also studied CKD severity classification, they used laboratory data as predictive features, including urine albumin, pH, lactade, sodium, and potassium levels, among other clinical measurements. Similar to our proposed method, Thakur et al. [29] used computer vision-based features extracted from a urine-based test strip and compared LR, SVM, and RF models, and ultimately found the RF model outperformed the alternatives. We designed our state-of-the-art baselines based on the methods proposed by Wei et al. [43], Ogunleye and Wang [59] and Thakur et al. [29].

Our SPR model achieved a *F1*-score of **0**.**9422**, which is substantially better than the best state-of-the-art approached we tested, and even outperformed a convolutional neural network-based model we designed. Moreover, we showed that SPR outperforms state-of-the-art baselines even when those baselines use the same color space as SPR rather than the original color space chosen by the authors. SPR uses a two-phase inference process, first selecting a local regressor best suited to predict the sample’s creatinine concentration, and then applying that regressor to make the prediction. The final kidney disease severity decision is made by thresholding on the estimated glomerular filtration rate (eGFR) value computed from the predicted concentration for the given sample. SPR is extremely versatile, allowing the use of any classifier or regressor as sub-estimators. Future novel classification and regression algorithms, as well as additional data collection will allow improving the effectiveness of the sub-estimators even further, which will improve SPR’s overall accuracy. In a series of ablation studies, we also showed that SPR is robust to meta-parameter choices, performing well under a variety of parameter choices, as long as enough data exist to properly train the chosen local regressors.

This study has a number of limitations. Overall, the image dataset was relatively small, even after sample augmentation, which may affect the performance of our machine learning models. Images were captured in a lightbox under controlled illumination and focus conditions, which are not representative of real images that a patient may capture at home using their smartphone. Since the machine learning models were trained with a relatively small dataset, these choices were justified by the need to minimize the confounding factors (such as focus, light brightness and hue). Notably, in our experiments we used synthetic creatinine solutions instead of blood. This choice removed the potential issue of hemoglobin interfering with the coloration of the detection pad, thus making it possible to use a simple LFA strip design. Moreover, using synthetic creatinine solutions allowed for full control over the creatinine concentration levels in the test samples.

The findings of this study are encouraging, and warrant further investigation. In the future, sample size can be increased by the implementation of a semi-automatic pipeline for data collection, currently under development in our laboratory. In addition, further refinement of the test strip, to include a stage for the separation of blood cells from serum, will enable tests with actual blood. We also plan to address potential inefficiencies in our model due to illumination inconsistencies in real-world image captures by integrating color constancy pre-processing in our image capturing pipeline.

### Supplementary Information

Below is the link to the electronic supplementary material.

Supplementary material 1 (PDF 381.4 kb)

## Data Availability

Data and open-source code for this study are available at https://github.com/davidanastasiu/spr.
