# Supplementary material for: Selective Partitioned Regression for Accurate Kidney Health Monitoring
Source: Ann Biomed Eng. 2024 Feb 27;52(5):1448–62. doi: 10.1007/s10439-024-03470-8 (PMC10995075; doi:10.1007/s10439-024-03470-8)
Supplement: Supplementary file 1 — Supplementary material 1 (PDF 381.4 kb) [file 10439_2024_3470_MOESM1_ESM.pdf]

Supplemental Materials (Not to be Published)

Selective Partitioned Regression for Accurate Kidney Health Monitoring
Technical Appendix

Alex Whelan, *Computer Science and Engineering, Santa Clara University*
Ragwa Elsayed, *Biomedical Engineering, San José State University*
Alessandro Bellofiore, *Biomedical Engineering, San José State University*
David C. Anastasiu, *Computer Science and Engineering, Santa Clara University*

Abstract—This technical appendix complements our article with the same title, providing additional clarification and experimental results that could not be included in the main article due to lack of space. The article describes SPR, a Selective Partitioned Regression model for kidney health monitoring, and shows that SPR is both effective and efficient in predicting the severity of kidney disease.

1 Reaction Color Characteristics Over Time

The reaction of the creatinine in a sample with the picric acid solution in the test strip takes some time, resulting in a colorimetric change of the test strip detection zone. The color changes over some time, stabilizes, and eventually starts to loose vibrancy as the chemicals dry. Fig. 2 illustrates the probability density function for the saturation and value (brightness) channels of Hue-Saturation-Value (HSV) representations of the detection zone images. We observe that the distribution of pixel values changes with elapsed time. This indicates that, as time progresses from 2 to 22 minutes, a sample will become more vibrant due to Jeffe's chemical reaction taking place. As time progresses, the pixel distribution will shift as the solution is being absorbed by the conjugate pad and the LFA begins to dry. We also analyzed chromacity, i.e., the specification of color independent of luma or lighting influence, by visualizing the hue probability density function of the detection zone sample, for creatinine concentrations 2, 6, 10, and 40 mg/dL. Our results, which are illustrated in Fig. 1, indicate that hue values also vary with reaction time for a given sample, and, more importantly, as expected, vary with the amount of creatinine concentration applied to the test strip.

2 Optimal Time Sampling

The reaction of the creatinine in each sample with the picric acid solution in the test strip takes some unknown amount of time. As a result, when we executed the chemical experiments, we took pictures of each test strip at 2, 12, and 22 minutes after applying the creatinine solution. We then trained all our models on samples captured at different time points in order to find the optimal time after the start of the reaction for deciding the severity of kidney disease. In these initial experiments, we used pixel values from the RGB color space as features, which is a standard approach for image processing, and tested the performance of all baseline models as well as our DNN model. Fig. 3 shows the result of these experiments. The left figure shows the overall classification performance while the right figure shows the regression performance.

Results indicate that, for the majority of the methods, the 22 minute time point provided the best classification and regression performance. In general, the worst results were achieved at 2 minutes, implying that 2 minutes is not enough time for the chemical reaction to give accurate results. Further experiments were then executed only at this time point. Moreover, we chose to eliminate the Support Vector Machines and Decision Tree algorithms from contention in further experiments as their performance was inferior compared to the rest of the available methods.

3 Ablation Studies

3.1 Color Space Comparisons

The chosen color space may play a big role in the performance of our model, as some color spaces separate luminescence from color representation while others do not. To see how the choice of color space in feature extraction affects model performance, we tested our model and all baselines with HOC-based features from each of the RGB, YC_rC_b, HSV, and LAB color spaces. Fig. 4 shows the *F1*-score and *RMSE* results for all models under each of the four color spaces. Note that the color spaces for each model are sorted in increasing *F1*-score and decreasing *RMSE* score order, i.e., the best color space is always towards the right.

Overall results indicate that the LAB color space is beneficial for the classification task but the HSV color space is best for the regression task. We believe the reasoning for this phenomenon is due to the way colors are represented within the binned histograms. LAB encodes the chroma value across two channels (a^* and b^*), compared with HSV that only uses a single channel (hue) for the task. Similarly, the apparent illumination of the chroma or the lightness value is encoded by two channels for HSV (value and saturation), versus only one channel for LAB. We believe HSV is able to discriminate more fine-grained differences

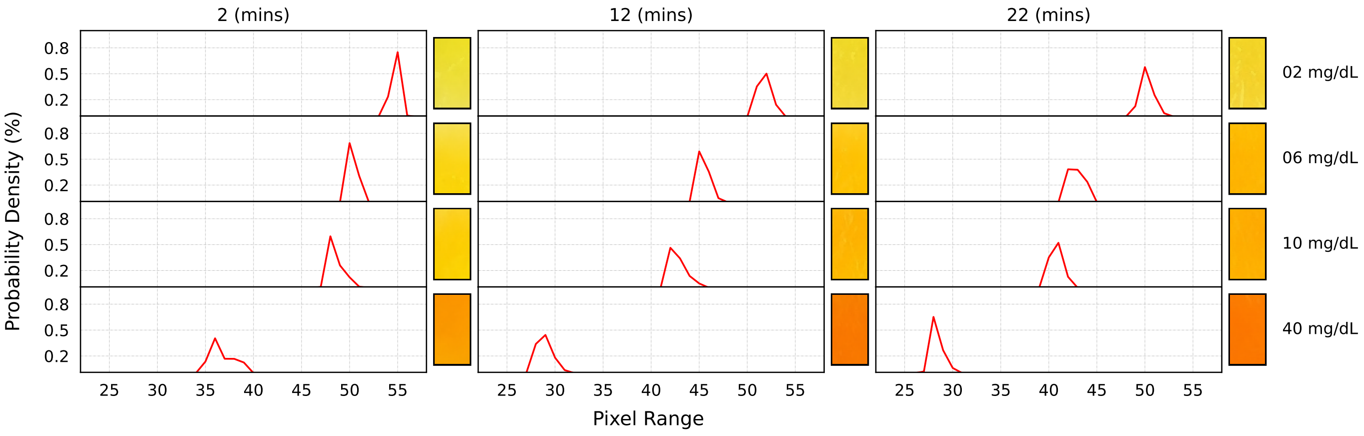

Fig. 1. Histogram visualization of the hue pixel density distribution for several collected samples with creatinine concentrations of 2, 6, 10, and 40 mg/dL. Each row shows the hue density distribution and detection zone image for the same sample after 2, 12, and 22 minutes from creatinine solution application.

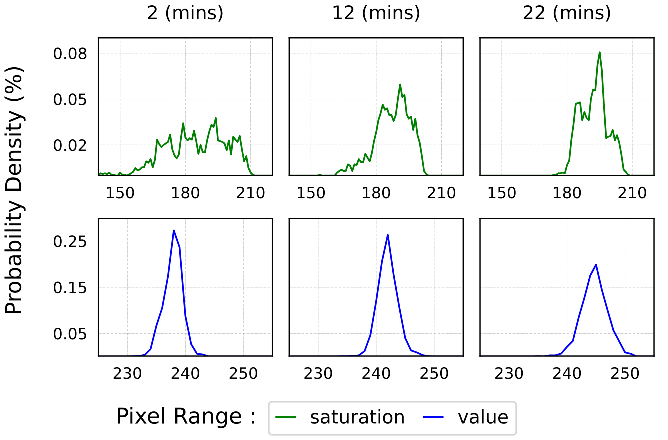

Fig. 2. Jeffe's chemical reaction affect on the probability density function for the saturation and value channels of a sample treated with 2 mg/dL of serum creatitine S_{Cr}.

in chroma values specifically within a certain bin concentration and LAB is able to more accurately differentiate course-grained concentration values, specifically ones at the classification boundaries. It is interesting to note, however, that the color space performance of our SPR model was consistent between the classification and regression tasks (RGB is worst and LAB is best), while other methods saw almost complete inversions between the two tasks (e.g., RGB achieves the highest/best *F1*-score for DNN and also the highest/worst *RMSE* score).

3.2 Feature Type Comparison

Another choice in our method is pixel- vs. HOC-based feature extraction. We analyzed the performance of the best performing baselines and our SPR model using both types of features and show the results in Fig. 5. The top chart shows *F1*-score values, while the bottom one shows *RMSE* values. Hatched bars show results for pixel-based models while the clear bars show HOC-based results. Finally, the color differentiates the models, as listed in the legend. Results clearly indicate that HOC-based features are superior for the majority of the models in both the classification and the regression tasks.

3.3 Partition Parameterization

Our model performance also depends on the number of local regressors χ and the bin ranges δ selected for each local regressor. In our initial experiments, we manually selected the bins to be equivalent to the ones we designed when performing the chemical experiments described in the main article, and extracted RGB color space pixel-based features. Subsequently, we tested both 3-bin and 4-bin configurations of our model with all color spaces and HOC-based features. For a configuration with P local regressors, one needs to choose $P-1$ points that define the concentration ranges the P regressors should be trained on. We used the finite set of 65 concentrations we defined during our chemical experiment design to search for optimal ranges via cross-validation. A complete grid-search for the 3-bin and 4-bin configuration would require roughly $2,112$ and 68K models to be trained and evaluated, respectively. We selectively chose 50 of the 65 concentrations and trained $1,250$ models for the 3-bin evaluations and we randomly chose $1,250$ configurations for the 4-bin evaluations.

Table 1 shows the optimum bin ranges identified for each configuration, along with the number of image samples (before augmentation) that belonged to each bin and the performance of our best SPR model for that configuration. The chosen color space is listed in parenthesis in the method name. Interestingly, while the 4-bin LAB-based configuration achieved the best CKD classification performance, the 3-bin HSV-based configuration achieved the best regression performance, as also noted in Section 3.1.

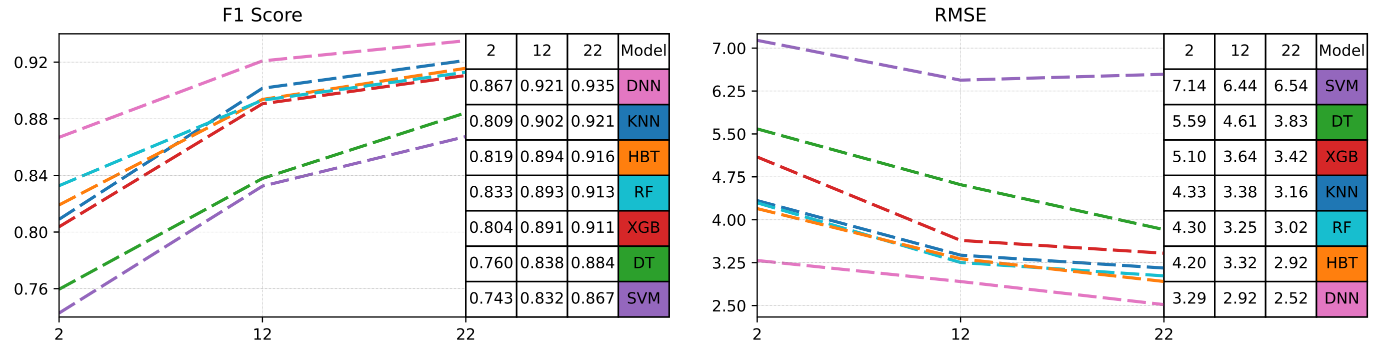

Fig. 3. Preliminary examination of baseline models using pixel-based features from the RGB color space at the three time points after the start of the reaction that images were captured at, namely 2, 12, and 22 minutes.

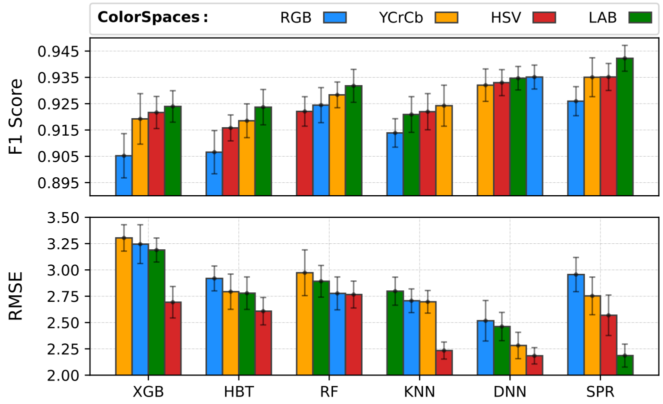

Fig. 4. Performance of models under various color spaces.

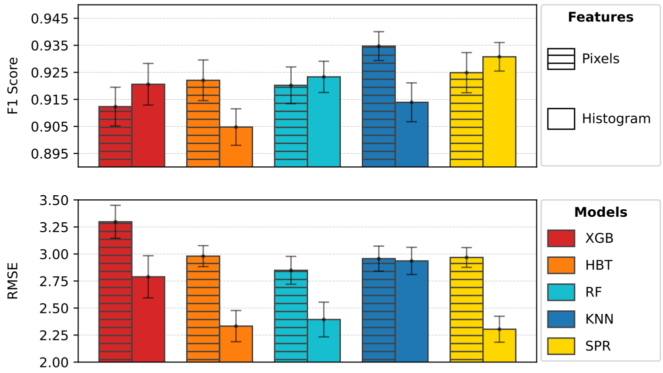

Fig. 5. Comparison of pixel- vs. HOC-based feature extraction from the LAB color space.

TABLE 1
Selective Partitioning Results

**Method**	**Range** (mg/dL)	**Bin**	**# Samples**	**F1**	**RMSE**
Default (RGB)	0.0 − 4.0	1	492	0.9234	2.74
	4.5 − 7.5	2	144		
	8.0 − 19.0	3	84		
	20.0 − 60.0	4	60		
3 Bin (HSV)	0.0 − 3.2	1	396	0.9316	**1.93**
	3.3 − 16.0	2	288		
	17.0 − 60.0	3	96		
4 Bin (LAB)	0.0 − 3.0	1	372	**0.9422**	2.19
	3.1 − 6.0	2	192		
	6.5 − 20.0	3	168		
	30.0 − 60.0	4	48		
